# Influences of Diet Quality, Nursery‐Habitat Complexity and Sex on Brain Development and Cognitive Performance of Brown Trout (*Salmo trutta* L.)

**DOI:** 10.1002/ece3.71924

**Published:** 2025-08-25

**Authors:** J. Peter Koene, Libor Závorka, Matthias Pilecky, Kathryn R. Elmer, Colin E. Adams

**Affiliations:** ^1^ Scottish Centre for Ecology and the Natural Environment (SCENE) University of Glasgow Glasgow UK; ^2^ School of Biodiversity, One Health and Veterinary Medicine University of Glasgow Glasgow UK; ^3^ WasserCluster Lunz – Biologische Station GmbH Lunz am See Austria; ^4^ Research Lab for Aquatic Ecosystems Research and Health Danube University Krems Krems Austria

**Keywords:** biosynthesis, cognition, encephalization, LC‐PUFA, routing, Salmonidae

## Abstract

Access to omega‐3 long‐chain polyunsaturated fatty acids (n‐3 LC‐PUFA) and habitat complexity have been proposed to influence brain development and cognitive ability. We aimed to investigate the physiological and cognitive effects of dietary n‐3 LC‐PUFA deprivation on juvenile brown trout ( *Salmo trutta* L.) in complex habitats resembling natal stream conditions in which populations have evolved. We tested effects of n‐3 LC‐PUFA deficiency in diet and habitat complexity on somatic growth, cognitive performance, encephalization, n‐3 LC‐PUFA biosynthesis and nutrient routing capacity. Brown trout were raised from egg for 7 months post‐hatch on either a high (8.91%) or low (1.79%) n‐3 LC‐PUFA diet; for the final 3 months, trout were further divided into complex (heavily ornamented tanks with small, dynamic, populations) or simple habitats (bare tanks with many, constant, inhabitants). Recognition, memory and inference were tested by comparing the times required to establish stable hierarchical relationships in agonistic dyadic trials featuring naïve trout and trials in which one of the trout had previously observed the other. Gas chromatography and compound‐specific stable hydrogen isotope analysis revealed increased biosynthesis and routing of n‐3 LC‐PUFA to the brain among trout on n‐3 LC‐PUFA‐deficient diets. Fed to satiation, trout did not sacrifice somatic growth to fuel biosynthesis and routing of n‐3 LC‐PUFA. However, dietary deficiency in n‐3 LC‐PUFA did lead to smaller brains, and smaller brains were associated with lower cognitive performance. Complex habitats elicited better cognitive performance, and were associated with lower somatic growth, but habitat complexity played only minor roles in encephalization and the n‐3 LC‐PUFA composition of brain lipids. We conclude that developmental plasticity in response to environment allows brown trout partially to compensate for the paucity of dietary n‐3 LC‐PUFA, and we suggest that cognitive divergences may play a role in the diversification of life‐history variants among brown trout in the wild.

## Graphical abstract

We demonstrate that variation in environmental inputs in early ontogeny can, in part, account for divergence in brain development and cognitive performance in brown trout. We also unveil a partial compensatory mechanism to address nutrient deprivation through bioconversion and routing of crucial omega‐3 fatty acids to brain membranes.

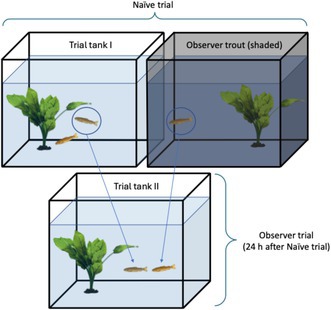

## 1 Introduction

Collectively, the processes of perception, learning, and memory that permit decision making constitute cognition (Shettleworth 2010a). Cognitive performance impacts the fitness and welfare of animals by influencing their capacities to forage, find potential mates, and avoid predators (Boogert et al. 2018; Fuss 2021). To collect and process the multitude of sensory inputs from their surroundings, many animals have evolved sophisticated brains that allow them to distinguish between predator and prey, assess competitors, discern their own movements through their environments, evaluate risk, and decide whether and where to hide (Fernö et al. 2020). Teleost fishes are particularly interesting for neurocognitive studies because, unlike other vertebrates such as mammals or birds, they continue even as adults to demonstrate neurogenesis throughout the entire brain (Folgueira et al. 2004a, 2004b, 2012).

Although not yet well studied, brain development has been found in some fish species to be reliant upon the quality of their diet during larval stages (Hou and Fuiman 2020). In particular, omega‐3 long‐chain (≥ 20 C) polyunsaturated fatty acids (n‐3 LC‐PUFA), especially docosahexaenoic acid (DHA 22:6n‐3), are important for neural development, structure, and function in vertebrates (Pilecky et al. 2021; Twining et al. 2021; Závorka et al. 2023). Because vertebrates have limited capacity to biosynthesize n‐3 LC‐PUFA, they must be acquired, at least in part, via the diet (Twining et al. 2016). Laboratory studies have shown a positive association between n‐3 LC‐PUFA enrichment and brain growth in freshwater and marine fishes (Lund et al. 2012; Ishizaki et al. 2001). In many animals, including fishes, cognition has been linked to relative brain size (Kotrschal et al. 2013; Triki et al. 2021). Brain size is associated with fitness‐influencing behaviours such as prey capture (Edmunds et al. 2016) and predator avoidance (Kondoh 2010). However, fish brain morphology varies widely across species (Triki et al. 2021), and so relative brain size alone may not always be the best proxy for fish cognition (Marhounová et al. 2019; Závorka, Lovén Wallerius, et al. 2022). In addition, different cognitive skills are associated with the relative sizes of specific brain regions: for example, learning and cognitive flexibility in response to visual stimuli, which are often investigated in animal cognition studies, are regulated by the telencephalon and optic tectum in guppies, *Poecilia reticulata* (Triki et al. 2022). Therefore, there is a need for experimental work integrating the impacts of diet quality on brain size, morphology, and biochemical composition, and the cognitive and behavioural abilities they promote.

For stream‐dwelling fishes, the combination of prey from freshwater aquatic and terrestrial origins provides for a varied dietary intake of essential nutrients (Sánchez‐Hernández and Cobo 2018; Syrjänen et al. 2011). Of the invertebrates typically consumed by juvenile salmonids, freshwater prey tends to be relatively rich in a n‐3 LC‐PUFA, eicosapentaenoic acid (EPA 20:5n‐3), which can be converted to DHA at relatively low metabolic cost, while prey of terrestrial origin tends to be richer in the short‐chain precursor, α‐linolenic acid (ALA 18:3n‐3), which can only be converted to DHA at high metabolic cost (Pilecky et al. 2021; Twining et al. 2021), which may lead to reduced somatic growth (Tocher 2010; Závorka et al. 2021). Furthermore, because n‐3 and n‐6 precursor fatty acids compete for the same enzymes, both synthesis pathways from α‐linolenic acid (ALA 18:3n‐3) to docosahexaenoic acid (DHA 22:6n‐3) and linoleic acid (LIN 18:2n‐6) to arachidonic acid (ARA 20:4n‐6) should be considered when investigating bioconversion of DHA precursors (Geiger et al. 1993): biosynthesis of ARA is, *ipso facto*, powerful evidence of even greater n‐3 LC‐PUFA conversion than what may be deduced from DHA alone (Sprecher 2000; Hastings et al. 2001).

In response to environmental conditions during ontogeny, developmental plasticity can expedite individual modification to morphological, behavioral, and physiological traits, with potential impacts to fitness in later life (Taborsky 2017; West‐Eberhard 2003). Greater environmental complexity or enrichment has been seen to induce increases in brain size and cognitive ability in fish (Ebbesson and Braithwaite 2012; Salena et al. 2021): evidence of plasticity‐inducing effects of habitat complexity on relative brain size exists for related salmonids, chinook salmon *Oncorhynchus tschawytscha* (Kihslinger et al. 2006) and Atlantic salmon *Salmo salar* (Näslund et al. 2012), as well as brown trout (Závorka, Lovén Wallerius, et al. 2022). Diverse environmental cues have also been shown to influence foraging and hierarchical social behavior, including risk‐taking, in salmonid species (Ayllón et al. 2025; Roberge et al. 2008; Thomson et al. 2012). Most of the conversion of short‐chain to long‐chain fatty acids occurs in the liver, from which DHA is distributed to the brain (Rapoport et al. 2007; Wang et al. 2017), where it may be incorporated into polar lipids (PL, mainly phospholipids) that increase neuronal membrane fluidity and accelerate the formation of synapses and synaptic vesicles as vehicles for neurotransmitters (Pilecky et al. 2021). DHA and its precursors that are not currently needed for a specific physiological function are stored in the form of neutral lipids (NL, mainly triacylglycerols) or allocated to other tissues, such as gonads or gametes for the next generation (Rigaud et al. 2023; Závorka et al. 2023; Zhu et al. 2019). Uneven distribution of DHA and its precursors, whether biosynthesized or dietary, may be caused by activation of storage lipids and rerouting of the required fatty acids to the brain (Lacombe et al. 2018; Pifferi et al. 2021). The combination of biosynthesis and priority routing of DHA and its precursors may represent a compensatory mechanism for those animals with diets depleted in such nutrients.

Brown trout *Salmo trutta* are a widespread species throughout Europe and introduced worldwide that exhibit enormous genetic, morphological, and life‐history variation and sexual dimorphism (Klemetsen 2013; Koene et al. 2020; Reyes‐Gavilán et al. 1997). Most variants typically are born and spend their early years as juveniles in low‐productivity streams (Ferguson et al. 2019), which offer both physical habitat complexity and instability (Guo et al. 2018) and the opportunity for social complexity in the form of dominance hierarchies in response to competition for preferred microhabitats (Sloman et al. 2000). Juvenile brown trout often lack morphological features that correspond to competitive ability that allow them to assess one another at the onset of each potential dyadic conflict (*sensu* Kodric‐Brown and Brown 1984), beyond mere differences in size (Jacob et al. 2007). So, recognition of individual rivals, memory of previous confrontations, and inference of competitive ability become important cognitive competencies when a potential opponent's fighting capabilities can otherwise be assessed only through escalated contests (Drew 1993).

To test the effect of dietary quality on recognition, memory, and inference abilities that are crucial for juvenile brown trout during conflicts (Johnsson and Åkerman 1998; Grosenick et al. 2007; Shettleworth 2010b), we conducted an experiment in which juvenile brown trout raised in either complex or simple habitats and on either high or low n‐3 LC‐PUFA diets were subjected to a series of agonistic dyadic trials. We hypothesized that larger brains, generally, larger optic tecta and telencephala, specifically, and greater DHA content of brain polar lipids are associated with greater cognitive performance; and that habitat complexity and dietary DHA deficiency stimulate increased compensatory DHA biosynthesis and routing to brain polar lipids, the energetic cost of which is reflected in lower somatic growth. Conducting a 2 × 2 experimental paradigm, we tested the predictions that: (1) trout deprived of n‐3 LC‐PUFA and in a complex habitat would show lower somatic growth than those raised on a high n‐3 LC‐PUFA diet and in a simple habitat; (2) trout raised on a high n‐3 LC‐PUFA diet and in a complex habitat would show greater cognitive performance than those raised on a low n‐3 LC‐PUFA diet and in a simple habitat; (3) trout raised on a high n‐3 LC‐PUFA diet and in a complex habitat would have larger relative brain size, and larger optic tecta and telencephala, than those raised on a low n‐3 LC‐PUFA diet and in a simple habitat; (4) that larger brains, and larger optic tecta and telencephala, would be associated with greater cognitive performance; and (5) trout deprived of n‐3 LC‐PUFA and in a complex habitat would biosynthesize DHA, and route DHA to brain polar lipids, to a greater extent than those raised on a high n‐3 LC‐PUFA diet and in a simple habitat.

There has been a suggestion that sex can play a role in the routing and synthesis of n‐3 LC‐PUFA in fish, with female Eurasian perch *Perca fluviatilis* showing significantly higher proportions of n‐3 LC‐PUFA in muscle tissue (Scharnweber and Gårdmark 2020), and rates of n‐3 LC‐PUFA biosynthesis before spawning (Rigaud et al. 2023), than males. Although sex differences are normally unexpected in brown trout until they reach maturity (Reyes‐Gavilán et al. 1997), we considered the potential impact of sex in all of our analyses.

## 2 Materials & Methods

### 2.1 Ethics Statement

All licensed experimental work involving live specimens was carried out under UK Home Office Licence No. PPL 70/8794.

### 2.2 Fish and Rearing Environment

Approximately 1000 late‐stage ‘eyed’ brown trout, *Salmo trutta* Linnæus 1758, eggs were obtained in early February 2021 from a stocking hatchery, AE Fishery, Moffat, UK. The eggs were the offspring of a mixture of riverine and lacustrine parents removed to captivity from the wild three to four generations ago. They were transported to the Scottish Centre for Ecology and the Natural Environment (SCENE) on Loch Lomond, UK, and approximately evenly distributed between 12 cylindrical 30 L flow‐through tanks held at *ca*. 4°C. Hatching was complete within 2 weeks of arrival at the centre.

At first feeding, *ca*. 10–14 days post hatch, to ensure that differences between individuals in aggression, swimming abilities, etc. did not affect group‐level results in later behavior trials, alevins‐turned‐fry were randomly split into two diet groups and moved to bare flow‐through tanks as above, six replicates per diet group, but without temperature control. Ambient water temperature naturally warmed throughout the growth season from *ca*. 4°C–21°C. Two diet types of near‐identical nutritional value were specially prepared by Garant‐Tiernahrung GmbH (Pöchlarn, Austria): one diet was high in n‐3 LC‐PUFA (EPA = 3.69%, DHA = 4.95%); the other diet was deficient in n‐3 LC‐PUFA (EPA = 0.9%, DHA = 0.89%) (Appendix A, Table A1). Fry were fed to satiation twice daily for *ca*. 4 months.

**FIGURE 1:**
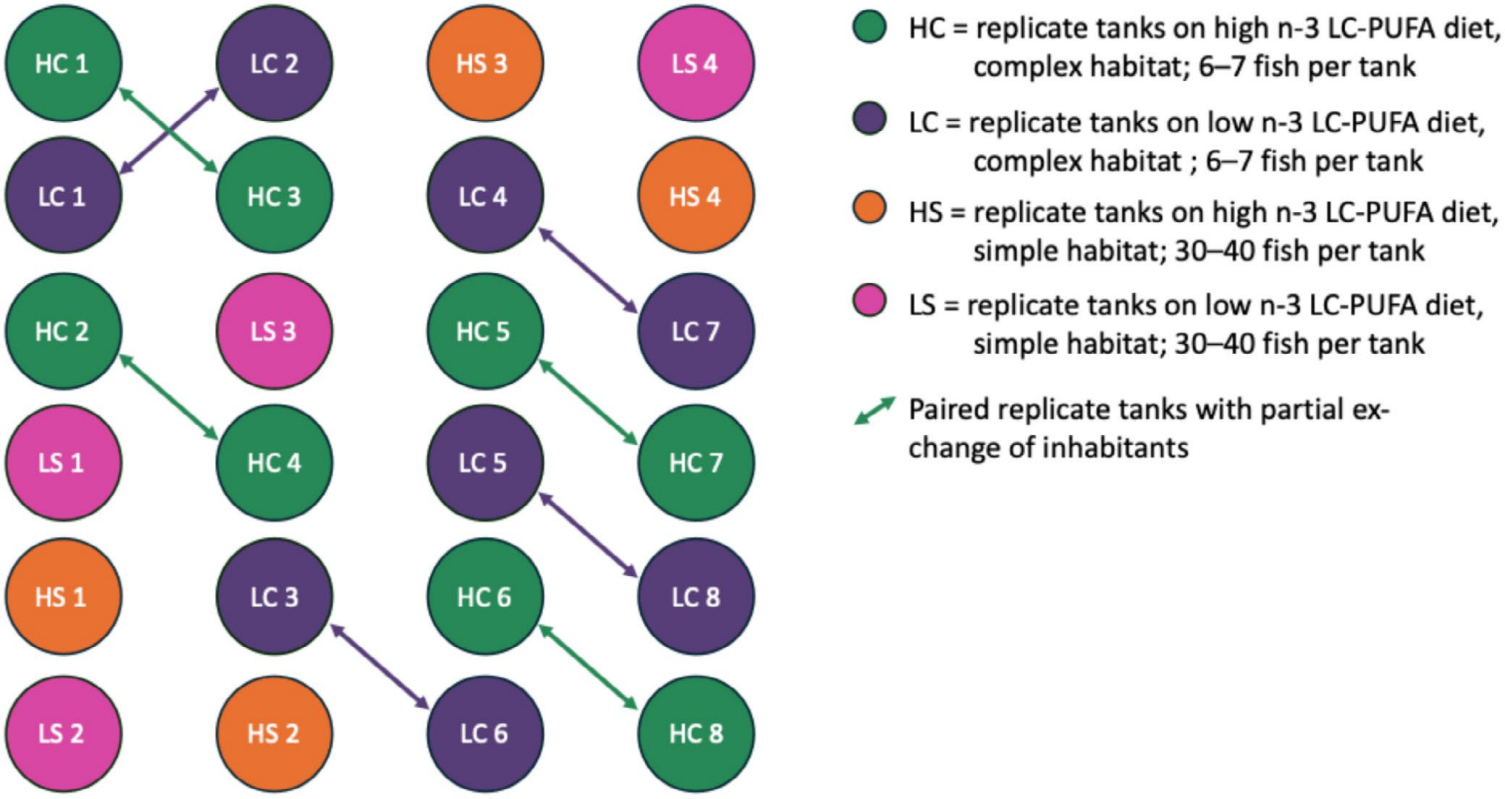
Schematic of treatment group replicates at the final stage of rearing (i.e., final 12 weeks), with the number of fish per tank, and indicating paired replicates of complex habitat tanks, in which partial exchanges of inhabitants occurred.

For the final 12 weeks of rearing, trout from each diet treatment were further randomly divided between ‘simple’ and ‘complete’ habitats. Simple habitats consisted of bare flow‐through cylindrical 90 L tanks with water supplied directly from Loch Lomond at ambient temperatures; 30–40 individuals per tank approximated conventional hatchery densities, which have been shown to be socially simpler than natural densities by inhibiting the development of competitive behaviors (Brockmark and Johnsson 2010). Complex habitats used the same tank type but were heavily ornamented. Ornament positions were altered every second day to simulate a dynamic environment. Tanks each housed 6–7 fish, and weekly part‐exchanges of fish between pairs of replicates fostered social complexity by allowing the continuous formation and reformation of dominance hierarchies (Sloman et al. 2000). All trout remained on their original diets and were, for the final 12 weeks, fed once daily to satiation, giving four distinct treatment groups, from which selections were made for further analysis: simple habitat, high n‐3 LC‐PUFA diet (four replicates); simple habitat, low n‐3 LC‐PUFA diet (four replicates); complex habitat, high n‐3 LC‐PUFA diet (eight replicates organised as four pairs part‐exchanging inhabitants); and complex habitat, low n‐3 LC‐PUFA diet (eight replicates organised as four pairs part‐exchanging inhabitants) (Table 1; Figure 1). At all stages of rearing, environmental variables (lighting, temperature, flow rate) were standardized across all tanks.

**TABLE 1:** Treatment groups and replicates at three stages of experimental rearing period of captive brown trout.

Stage	Duration in weeks	Treatment	Replicates	Fish per replicate	Feedings per day
Eggs & alevins	2	Common	12	~80–85	0
Fry in diet groups only	16	High	6	~50	2
		Low	6	~50	2
Fry in habitat and diet groups	12	High, simp.	4	~35	1
		Low, simp.	4	~35	1
		High, comp.	8	6–7	1
		Low, comp.	8	6–7	1

Abbreviations: comp., complex habitat; High, high n‐3 LC‐PUFA diet; Low, low n‐3 LC‐PUFA diet; simp., simple habitat.

### 2.3 Behavioural Trials

Following the final 12‐week rearing period, all trout were anaesthetised with a solution of benzocaine, measured between the tip of the snout and the fork of the tail (i.e., ‘fork length’: 71 ± 8 mm, mean ± SD), and marked with a pattern of variously coloured visible implant elastomer tags (Northwest Marine Technology Inc., Anacortes, WA, USA). Sixty groups of three trout each were established. Within each triad, trout were size‐matched, in which the largest was larger by < 5% of the fork length of the smallest, to minimise possible effects of size on dominance (Huntingford et al. 1990; Johnsson and Åkerman 1998). Additionally, individuals were unfamiliar with one another, having never shared a rearing tank. Each triad consisted of a dyad from different replicates of a treatment group, plus an ‘observer’ from another replicate. Across the 60 triads, all combinations of replicates and treatment groups were represented with the same approximate frequency. The sample size allowed for approximately 30 individuals per grouping by diet, habitat, and sex for downstream analyses. This accorded with power analysis assuming a moderate effect size of 0.35, significance level of 0.05, and power of 0.75.

**FIGURE 2:**
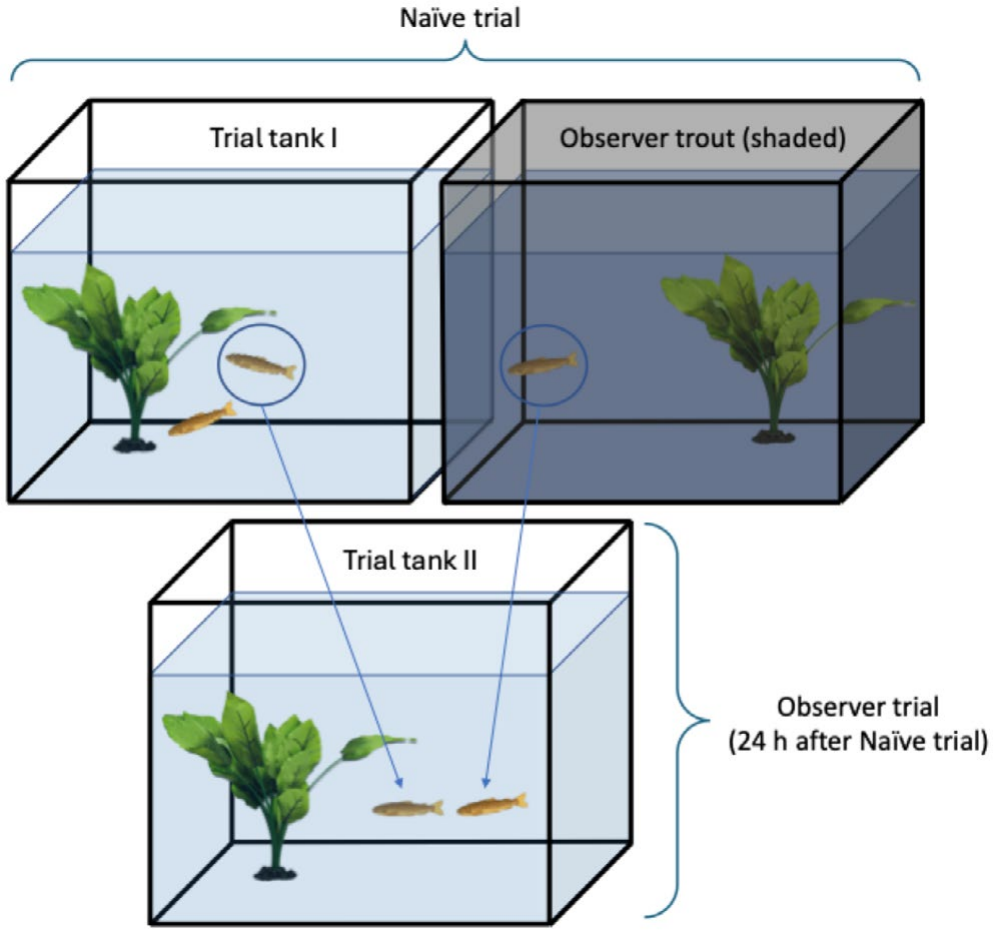
Aquarium set for behavioral trials of a trout triad: The naïve trial (Trial tank I) shows a trout dyad establishing a dominant/subordinate relationship while a third trout observes unseen (upper right tank). The observer trial (Trial tank II) shows the former observer and one of the previous dyad establishing a new dominant/subordinate relationship. Visual blockages on all sides, except that facing the researcher and between the naïve trial tank and the observer trout, are not shown here.

Behavioural tests were held as dyadic trials in successive stages between groups of three. Triad members were kept alone and separate from one another before and between behavioural trials in identical 30 L glass aquaria, adorned with an air stone and one plastic plant set in a corner, for 24 h and fasted. For the initial behavioural trials (*naïve trials*), a dyad was placed simultaneously into a replica of the fasting aquaria. This trial tank was open at the top and illuminated by a 26 W, 1750 lm ceiling lamp. The sides were visually blocked, except the front, to allow observation by the researcher, and one side, to allow observation by the adjacent tank inhabitant. In an identical aquarium adjacent, the third trout of the size‐matched triad was given the opportunity to observe the first two. This aquarium was shaded to prevent the dyad from observing the observer, and it was visually blocked on all sides except that facing the trial tank (Figure 2).

The dyad was observed by a researcher for 5 min every half hour until a dominant/subordinate relationship was seen to be clearly established, or until 5 h had elapsed (*vide* Appendix A). Dominance and subordination were assessed by assigning points at each observation using a point‐scoring system that evaluated levels of concealment, activity, and aggression (Table 2), based on a previous study of brown trout behavior (Sloman et al. 2000). A stable dominant/subordinate relationship was *suspected* if one fish had a positive score that was at least two points higher than the other fish's. At the next observation following suspicion of a stable relationship, two single bloodworms dropped simultaneously were introduced at the center of the forward‐facing glass panel. If the suspected dominant fish took the food first and retained a two‐point or greater score advantage, a dominant/subordinate relationship was *declared*. Otherwise, the relationship was considered unsustained. At the observation following declaration, the food drop was repeated, and if the same results were seen, the relationship was considered confirmed as stable, and the time of the declaration recorded. Without confirmation, a declaration of a stable relationship was voided. All trials were observed by the same researcher. After the naive trials, trout were fed, then returned to their individual fasting tanks for 24 h.

**TABLE 2:** Scoring system used at repeated observations to detect the establishment of a dominant/subordinate relationship between pairs of brown trout in behavioural trials.

Behaviour	Description	Score at each observation
Concealment	Hiding	0
	Not hiding	1
Activity	Active avoidance	−1
	Resting (inactive)	0
	Swimming in water column	1
Aggression	Victim	−1
	No aggression	0
	Rubbing against, or quickly darting at, other	1
	Extended chasing and/or nipping other	2
Feeding	No food taken	0
	Second to take food	1
	First to take food	2

A second behavioural trial (*observer trial*) was conducted 24 h after the naïve trial. This tested the observer trout's ability to establish a dominant/subordinate in less time than the original dyad, indicating greater cognitive performance. This was based on the premise that a trout that can recognise a con‐specific it has previously observed, remember something of its attributes, and infer how its own attributes compare, will more quickly adopt a stable social position, either dominant or subordinate, than it would if presented with an unknown trout (Drew 1993). The previous day's observer trout was placed into a new trial tank with one from the original dyad, which alternated between initially dominant or subordinate with each trial replicate. Apart from the absence of a trout in an adjacent tank overlooking the dyad, the observer trial followed the same procedure as the naïve, with the former observer now as an active dyad participant. To avoid interobserver error, all trials were scored by a single researcher.

### 2.4 Sexing by Genotype

Upon completion of the observer trials, all trout were euthanized with an overdose of benzocaine solution. Genomic DNA was extracted from adipose fin clips using a NucleoSpin Tissue kit (Macherey‐Nagel GmbH & Co. KG, Düren, Germany) following the manufacturer's instructions; quality controlled with spectrophotometry (NanoDrop, ThermoFisher Scientific, Waltham, MA, USA); and quantified fluorometrically (Qubit 2.0, ThermoFisher Scientific, Waltham, MA, USA). Extracted DNA was diluted to 20 ng μL^−1^.

To determine the sex of each trout, following a modification of established protocols (Anglès d'Auriac et al. 2014; Lavender et al. 2024), duplex PCR amplified the male‐specific Y‐chromosome gene, *sdY* (forward primer: CCC AGC ACT GTT TTC TTG TCT CA; reverse primer: CTT AAA ACC ACT CCA CCC TCC AT), using the *18S* gene as a positive amplification control (forward primer: GTY CGA AGA CGA TCA GAT ACC GT; reverse primer: CCG CAT AAC TAG TTA GCA TGC CG). PCR was performed using 3 μL of DNA with 0.3 μL of each *sdY* primer, 0.075 μL of each *18S* primer, 7.5 μL of Qiagen Multiplex PCR mix containing 3 mM MgCl_2_, HotStarTaq DNA polymerase, and proprietary buffer (Qiagen N.V., Hilden, Germany), and 2.2 μL nuclease‐free H_2_O. Thermal cycling consisted of initialization for 15 min at 95°C, followed by 35 amplification cycles of 30 s at 94°C, 90 s at 63°C, and 90 s at 72°C, with a final extension for 10 min at 72°C. The resulting PCR products were visualized with 2% agarose gel electrophoresis.

### 2.5 Encephalization and Brain Morphology

**FIGURE 3:**
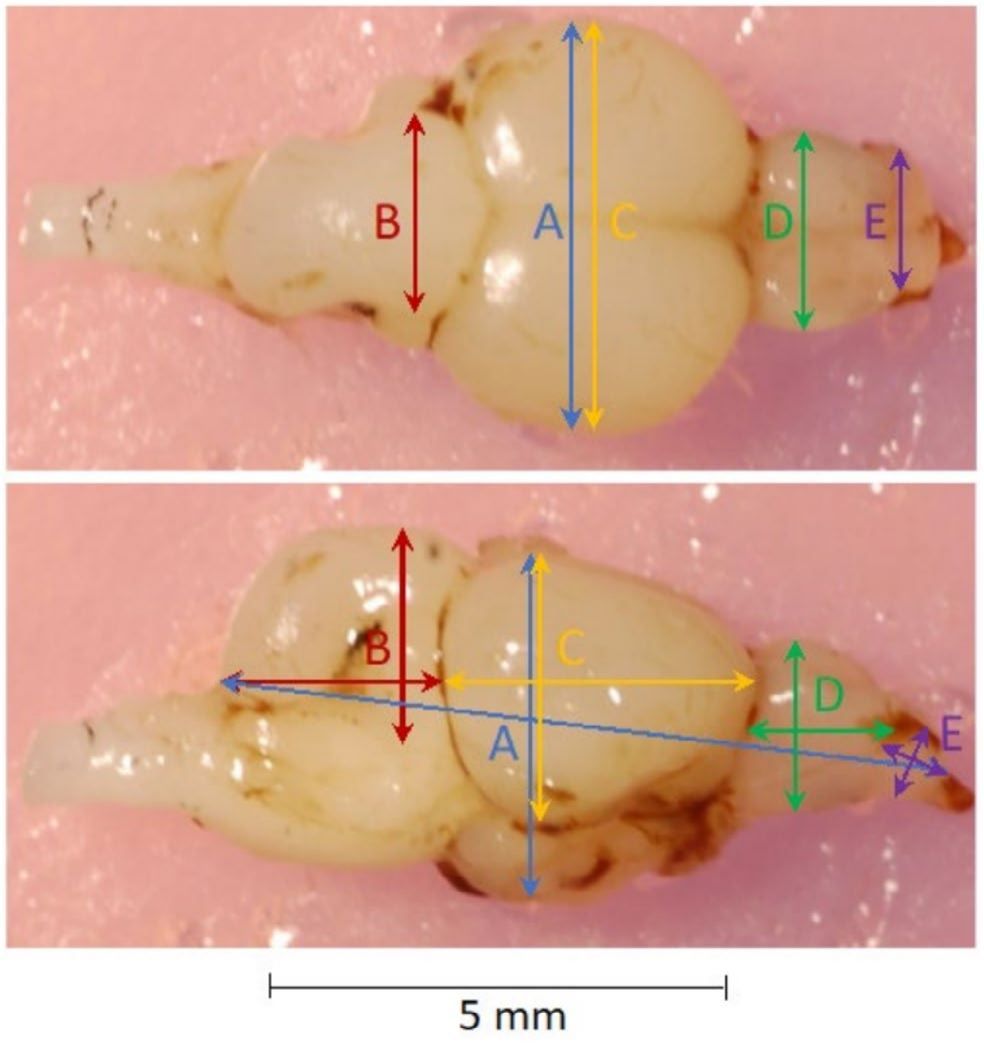
Dorsal and lateral views of a brown trout brain, showing width, length and depth measurements of (A) whole brain, (B) cerebellum, (C) optic tectum, (D) telencephalon, and (E) olfactory bulb.

To standardize dissection and measurement procedures, all were performed by a single researcher. Heads of trout from half the trial triads (i.e., 90 individuals), representing all treatment groups, were removed and fixed for 24 h in 4% buffered (pH 6.9) paraformaldehyde solution. Brains were removed following the procedure described by Závorka, Koene, et al. (2022) and fixed for a further 24 h in the buffered paraformaldehyde solution. They were then weighed to the nearest 0.1 mg. Dorsal and right lateral views of brains were photographed with a Nikon D50 DSLR camera (Nikon Corporation, Tokyo, Japan) and Sigma 70 mm F2.8 DG Macro lens (Sigma Corporation, Tokyo, Japan). Measurements were made using Image J 1.48 (Schneider et al. 2021) of the length (L), width (W) and depth (D) of the whole brain and, independently, of cerebellum, optic tectum, telencephalon, and olfactory bulb (Figure 3). Volumes (V) were calculated for whole brains with a corrected ellipsoid formula. To temper the systematic overestimation of volume due to deviations of brain shape from ellipsoid, a correction factor was introduced, following Pollen et al. (2007), using the slope of the linear regression of uncorrected brain volume on actual brain mass (i.e., 1.022):

$$V=\left(L\times W\times D\right)\pi /\left(6\times 1.022\right)$$

Volumes of brain regions were calculated with the ellipsoid formula without the correction factor (Pollen et al. 2007). As measures of relative whole brain mass, residuals of a linear regression of actual whole brain mass on body mass were used. Similarly, volumes of each brain region were regressed on whole brain volume, and residuals were used as relative volumes of each brain region. On eight occasions during removal, brains were damaged (typically the delicate olfactory bulbs were severed); these samples were removed from subsequent analyses.

### 2.6 Gas Chromatography and Mass Spectrometry

Whole brains and samples of dorsal muscle tissue were removed immediately upon death from the observers of the remaining triads (i.e., 30 trout), also representing all combinations of treatment group, flash frozen in liquid nitrogen, then freeze‐dried and stored at −80°C to limit lipolytic degradation. The immediacy of this method of preservation was not compatible with the brain‐measuring procedure described above. Samples of food sources were included with tissue samples in subsequent fatty acid analyses.

Lipid extraction and esterification from freeze‐dried samples followed the protocol described by Pilecky, Fink, et al. (2023). Briefly, whole brains (*ca*. 10 mg) and *ca*. 30 mg of muscle and food source samples were weighed to the nearest 0.01 mg, homogenized, and stored in chloroform (2 mL) under N_2_ gas overnight at −80°C. With the addition of 1 mL MeOH and 750 μL of 0.9% NaCl solution, samples were repeatedly sonicated, vortexed, and centrifuged to remove non‐lipid materials. Solvent was fully evaporated from extracted lipids, and 2 mL chloroform was added under N_2_. Gravimetry of aliquots was performed as a measure of total lipids for each sample.

Neutral and polar lipids were separated using BondElut Ultra Inert GC columns (Agilent Technologies Inc., Santa Clara, CA, USA). Column equilibration was conducted by passing hexane through each column; then, 10 mg of lipids–chloroform solution were loaded. Neutral lipids (NL) were isolated with 4 mL of 2:1 chloroform: isopropanol and evaporated fully. Then, to remove free fatty acids, 4 mL of 2% acetic acid in diethyl ether was run through the columns and discarded. Polar lipids (PL) were subsequently eluted with 4 mL of methanol and evaporated fully.

Fatty acid methylated esters (FAME) of the extracted NL and PL were formed by incubation with 1% H_2_SO_4_ in MeOH for 16 h at 50°C, followed by the addition of 2 mL of 2% KHO_3_ and 2 mL hexane, following Pilecky, Wassenaar, et al. (2023). Samples were mixed and centrifuged, and the upper organic layer of each sample was collected and concentrated under N_2_ gas.

Gas chromatography (TRACE GC 1310, Thermo, Waltham, MA, USA) of FAME followed the protocol established by Pilecky, Fink, et al. (2023), using external standards for calibration; concentrations were reported as mg g^−1^ dry weight. Data involving candidate n‐3 and n‐6 FAME were carried forward for further investigation.

For fatty‐acid‐specific stable isotope ratio mass spectrometry (DELTA V Advantage, ThermoFisher Scientific, Waltham, MA, USA), the gas chromatograph was coupled via CONFLO IV (Thermo, Waltham, MA, USA). Samples were run against certified Me‐C20:0 stable isotope reference material (USGS70: *δ* ^2^H = −183.9‰, USGS71: *δ* ^2^H = −4.9‰ and USGS72: *δ* ^2^H = +348.3‰) and corrected for methylation, as described elsewhere (Pilecky, Wassenaar, et al. 2023). Food sources were used further to correct the *δ* ^2^H signature of each FAME in each sample:

$$\Delta \delta^{2}H_{\text{FAME}}=\text{sample}\;\delta^{2}H_{\text{FAME}}–\text{mean}\left(\text{food source}\;\delta^{2}H_{\text{FAME}}\right)$$

Depletion of Δ*δ* ^2^H in successive FAME in the biosynthesis pathways indicates bioconversion of shorter chain PUFA rather than a dietary source of LC‐PUFA (Pilecky et al. 2022).

### 2.7 Statistical Analyses

All statistical analyses were conducted in *R* v.4.2.2 (R Core Team 2022). To test for influences on somatic growth, the effects of diet, habitat (plus interaction), and sex on fork length for all trout (*n* = 180) were modeled with a linear model.

To test factors influencing the cognitive performance of observer trout, a linear model modeled the effects of diet, habitat (plus interaction), and sex on the difference in time for each triad between the observer trials and naïve trials (i.e., observer trial time minus naïve trial time) until the dominant/subordinate relationship was clearly established. Triads that were unable clearly to establish dominance/subordination in either the naïve or observer trial were omitted from this and subsequent models. To ensure that triads were appropriately size‐matched, linear mixed effects models using triad as a random effect confirmed that whether individuals would become dominant or subordinate was not affected by small differences in size after approximate size matching (naïve trail: *F*_1,104_ = 0.013, *p* = 0.911; observer trial: *F*_1,104_ = 0.06, *p* = 0.809).

To test factors influencing encephalization, the effects of diet, habitat (plus interaction) and sex on relative whole‐brain mass for all trout whose brains were preserved in formalin were modelled with a linear model. To consider brain morphology, MANOVA tested the effects of diet, habitat (plus interaction), and sex on relative volumes of brain regions. ANOVA followed to test the effects on brain regions individually. To test the effects of encephalization and brain morphology on cognitive performance, a linear regression modelled time differences between naïve and observer trials on relative brain mass and relative volumes of brain regions in addition to habitat and sex of those observer trout whose brains had been extracted intact (*n* = 52); diet was omitted as it was colinear with, and causally linked to, encephalization. To determine whether a relationship between brain mass and cognitive performance depended on sex, ANOVA was used to test the effect of sex on the residuals of a regression of relative brain mass on the time difference between trials.

To examine differences between treatment groups in how individual fatty acids were routed to specific tissue/lipid types (i.e., brain and muscle tissue, polar and neutral lipids), mean percentages of total lipids composed of individual fatty acids across tissue/lipid types were tested with one‐way ANOVAs followed by Tukey's HSD *post hoc*. To determine whether fatty acid contents of various tissue/lipid types were influenced by treatment, the effects of diet, habitat (plus interaction) and sex on the percentage of each fatty acid in the n‐3 and n‐6 bioconversion pathways were tested with MANOVA. Effects on each fatty acid percentage were then tested with ANOVA for individual tissue/lipid types. Differences between treatment groups in the depletion of Δ*δ* ^2^H in fatty acids across tissue/lipid types were evaluated by modeling the effects of diet, habitat (plus interaction) and sex on the Δ*δ* ^2^H of individual fatty acids and tested with MANOVA. For specific tissue/lipid types, effects on Δ*δ* ^2^H were tested with ANOVA.

## 3 Results

### 3.1 Growth, Encephalization and Brain Morphology

Across all trout in the study (*n* = 180), habitat and sex had small but significant effects on fork length: trout raised in simple habitats were larger than those raised in complex habitats (*F*_1,175_ = 6.71, *p* = 0.01), and males were larger than females (*F*_1,175_ = 5.06, *p* = 0.026). However, there were no significant diet or diet: habitat interaction effects (Table 3).

**TABLE 3:** Effects of diet, habitat and sex on fork length (FL) of 180 experimental brown trout, tested with ANOVA.

Factor	Level	Mean FL (mm)	SD	*F*_1,175_	*p*
Diet	High LC‐PUFA	71.3	8.2	3.35	0.553
	Low LC‐PUFA	70.7	8.4		
Habitat	Complex	69.4	9.4	6.71	0.010
	Simple	72.5	6.8		
Sex	Female	69.5	7.8	5.06	0.026
	Male	72.2	8.5		
Diet:Habitat	(interaction)			1.36	0.245

**FIGURE 4:**
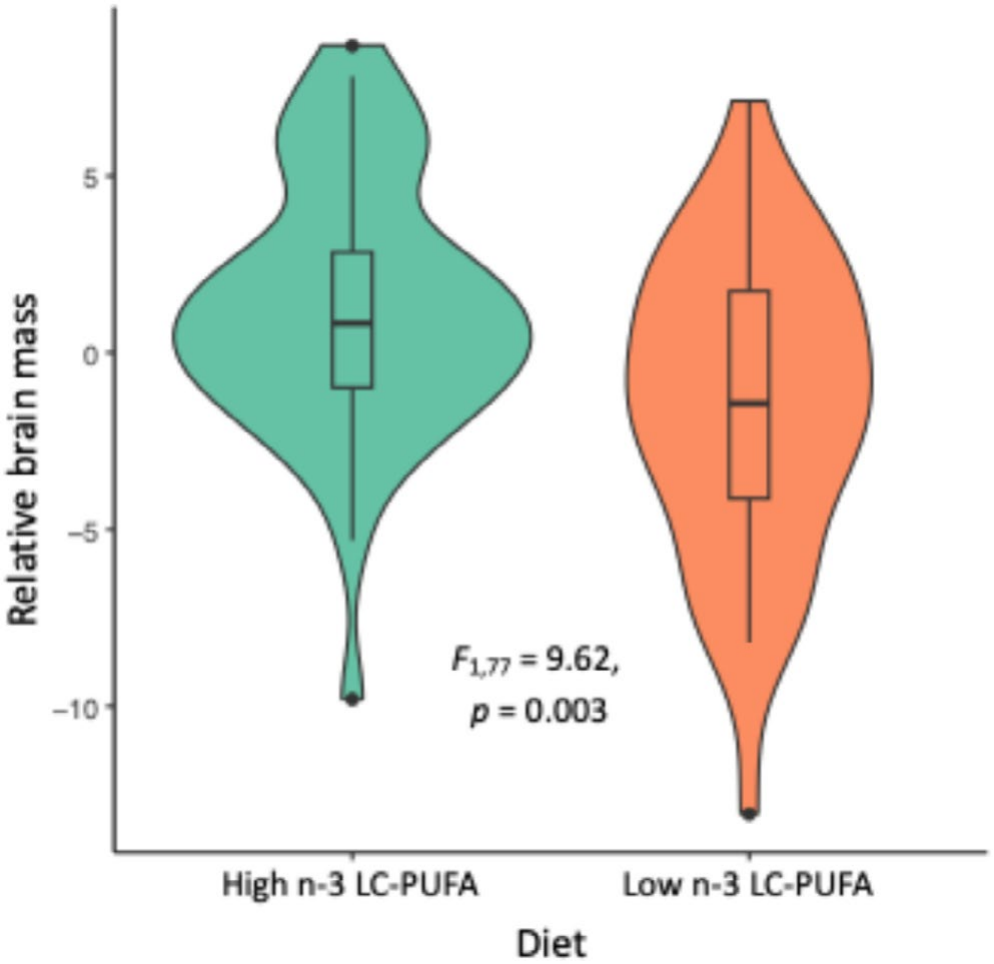
Effect of high and low n‐3 LC‐PUFA diets on relative brain mass (i.e., residuals of a linear regression of actual brain mass on body mass) of 78 experimental brown trout.

Among those subjects whose brains had been preserved for morphological analyses (*n* = 78), neither sex nor rearing habitat had a significant effect on relative brain mass, but those trout raised on the high n‐3 LC‐PUFA diet had larger brains than those raised on the low n‐3 LC‐PUFA diet (*F*_1,77_ = 9.62, *p* = 0.003) (Table 4; Figure 4). Although sex had no significant effect on the relative volume of any brain region, there was possibly an interaction between diet and habitat that did affect olfactory bulb size (MANOVA: Pillai = 0.12, *F*_1,77_ = 2.43, *p* = 0.055; ANOVA *post hoc*: *F*_1,77_ = 5.96, *p* = 0.017): in simple habitats, trout raised on low n‐3 LC‐PUFA had larger olfactory bulbs, while in complex habitats, it was those raised on high n‐3 LC‐PUFA that had the larger olfactory bulbs. No other brain region was specifically affected by diet or habitat.

**TABLE 4:** Effects of diet, habitat and sex on the relative brain mass (i.e., residuals of linear regression of actual brain mass on body mass) of 78 experimental brown trout, tested with ANOVA.

Factor	Level	Rel. brain mass	SD	*F*_1,77_	*p*
Diet	High LC‐PUFA	1.241	3.669	9.616	0.003
	Low LC‐PUFA	−1.509	4.273		
Habitat	Complex	0.386	5.076	0.801	0.374
	Simple	−0.350	3.135		
Sex	Female	−0.113	4.115	0.001	0.970
	Male	0.084	4.239		
Diet:Habitat	(interaction)			0.455	0.502

### 3.2 Cognitive Performance

**FIGURE 5:**
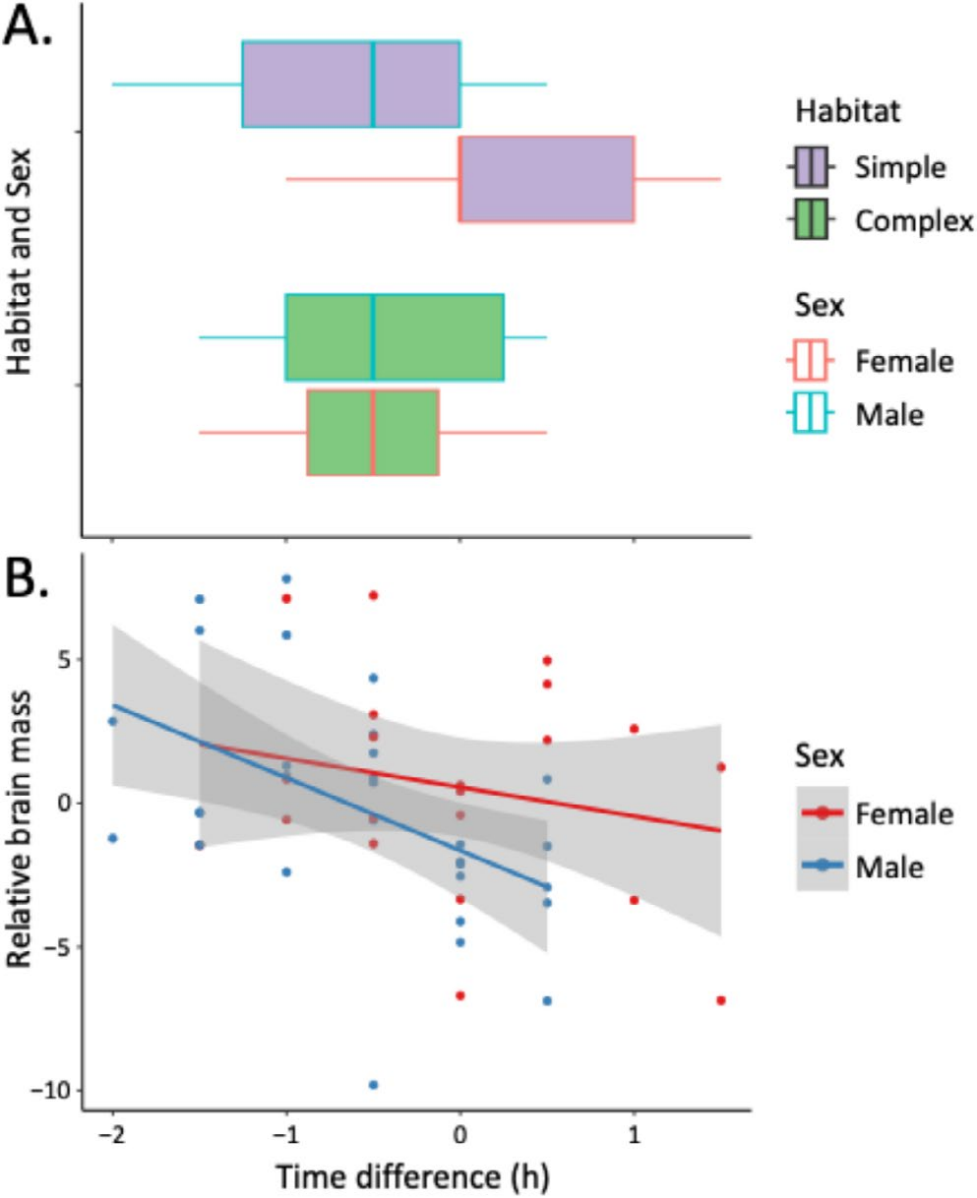
Effects of (A) habitat and sex and (B) relative brain mass on the difference between observer and naïve trials in time (i.e., observer trial time minus naïve trial time) required by trout triads to establish stable dominance/subordination.

In the observer trials, 55 of 60 pairs established clear dominant/subordinate relationships within the 5‐h period. Considering time differences between observer and naïve trials (i.e., observer trial time minus naïve trial time), trout raised in complex habitats showed greater improvement in time needed to establish a stable hierarchy in the observer trial than did those from simple habitats (*F*_1,98_ = 13.09, *p* < 0.001). Females generally showed less improvement than did males in the observer trials over the naïve trials (*F*_1,98_ = 4.57, *p* = 0.034), especially those from the simple habitat; but, females from the complex habitat showed greater improvement than did males (sex/habitat interaction: *F*_1,98_ = 11.41, *p* = 0.001) (Figure 5A). Greater brain mass was also associated with quicker times in the observer trial (*F*_1,42_ = 6.93, *p* = 0.011), an effect that was more pronounced in males than in females (*F*_1,49_ = 8.55, *p* = 0.005) (Figure 5B). However, no specific brain region exerted a significant effect on time differences between trials (telencephalon: *F*_1,42_ = 1.87, *p* = 0.179; optic tectum: *F*_1,42_ = 0.24, *p* = 0.627; cerebellum: *F*_1,42_ = 0.05, *p* = 0.828; olfactory bulb: *F*_1,42_ = 0.88, *p* = 0.353).

### 3.3 Fatty Acid Analyses

**FIGURE 6:**
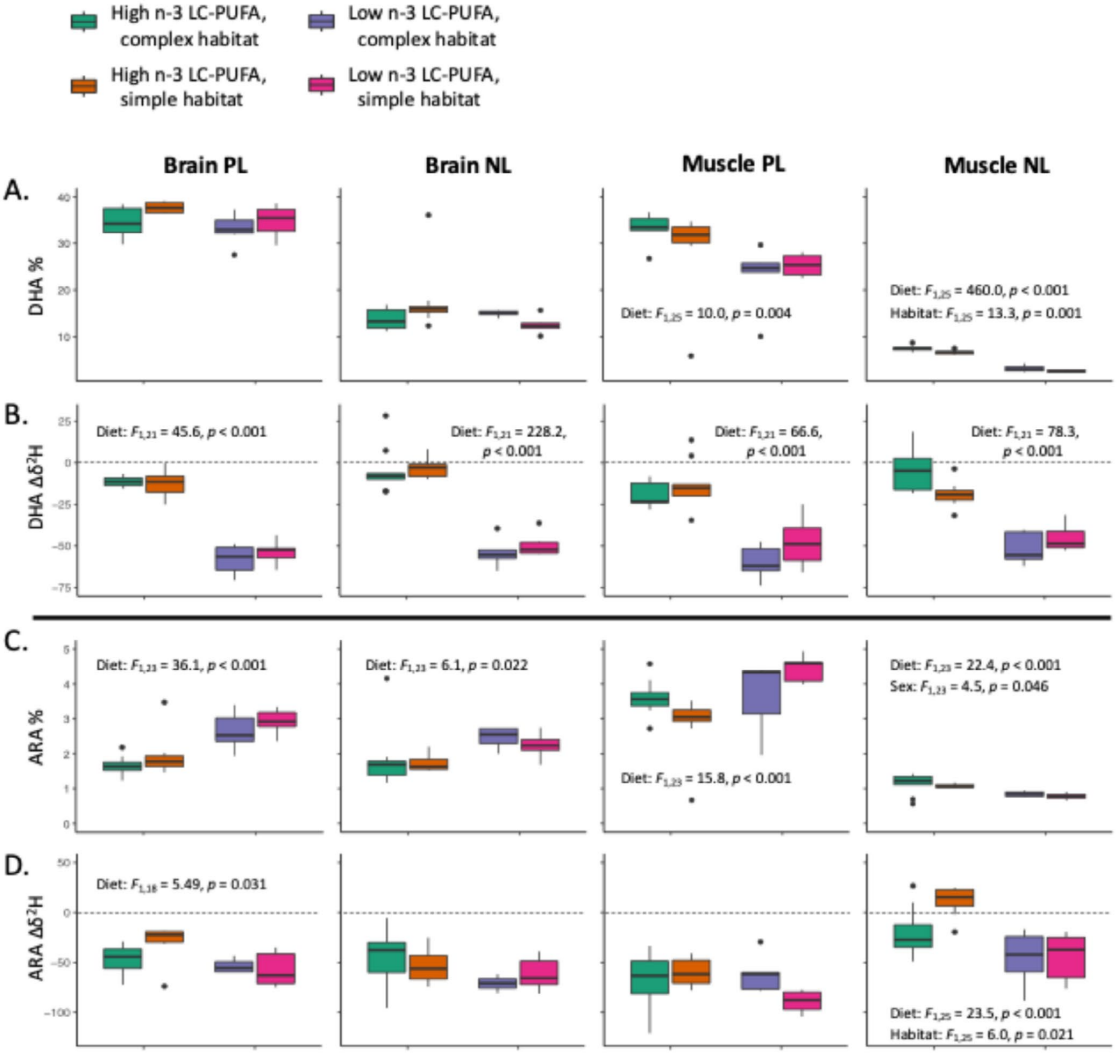
Effects of diet and habitat (and sex), for each of four tissue/lipid sample types, on (A) the percentage of total lipids composed of DHA 22:6n‐3, (B) Δ*δ* ^2^H_DHA_, (C) percentage ARA 20:4n‐6, and (D) Δ*δ* ^2^H_ARA_. Significant effects are noted. PL, polar lipids; NL, neutral lipids.

There were significant differences in DHA routing to various tissue/lipid types (*F*_3,114_ = 239.6, *R* ^2^_adj_ = 0.86, *p* < 0.001), with brain PL receiving the highest percentage (*post hoc*: brain PL compared to each other type, all *p* < 0.001) and muscle PL having a higher percentage than NL in either brain or muscle (*post hoc*: both *p* < 0.001) (Figure 6A; Appendix A, Table A2). For individual tissue/lipid types, however, there were differences in DHA content between diet groups (Pillai = 0.97, *F*_1,25_ = 153.3, *p* < 0.001) and habitats (Pillai = 0.38, *F*_1,25_ = 3.34, *p* = 0.027). Trout on the low n‐3 LC‐PUFA diet had lower DHA percentages than trout on the high n‐3 LC‐PUFA diet in muscle PL (*F*_1,25_ = 10.0, *p* = 0.004) and muscle NL (*F*_1,25_ = 460.0, *p* < 0.001), although there was no significant difference in brain lipids. Trout raised in complex habitats had a higher DHA percentage in muscle NL (*F*_1,25_ = 13.3, *p* = 0.001); habitat had no significant effect on routing DHA to other tissue/lipid types (Figure 6A; *vide* Appendix A, Table A2).

Depletion of ∆*δ* ^2^H_DHA_ indicated there was considerable biosynthesis among trout fed the low n‐3 LC‐PUFA diet (Pillai = 0.95, *F*_1,21_ = 80.6, *p* < 0.001), despite lower DHA content. The effect of diet on ∆*δ* ^2^H_DHA_ depletion was highly significant (all *p* < 0.001) for all tissue/lipid types (Figure 6B). Neither habitat nor sex exerted a significant effect on ∆*δ* ^2^H_DHA_ depletion (*vide* Appendix A, Table A3).

Because content and depletion of ∆*δ* ^2^H in the n‐6 LC‐PUFA, ARA 20:4n‐6, are related to n‐3 LC‐PUFA biosynthetic activity, it is important to consider these when investigating effects on DHA (Sprecher 2000). ARA content across tissue/lipid types was affected by diet (Pillai = 0.73, *F*_1,23_ = 13.52, *p* < 0.001), but not habitat or sex. Trout raised on the low n‐3 LC‐PUFA diet had significantly higher percentages of ARA than the trout raised on high n‐3 LC‐PUFA in each tissue/lipid type (from brain NL, *F*_1,23_ = 6.06, *p* = 0.023 to brain PL, *F*_1,23_ = 36.08, *p* < 0.001) (Figure 6C; *vide* Appendix A, Table A3). This was reflected in greater biosynthesis among the low n‐3 LC‐PUFA‐fed trout than the high n‐3 LC‐PUFA‐fed trout, seen in the greater depletion of Δ*δ* ^2^H_ARA_ in brain PL (*F*_1,18_ = 5.49, *p* = 0.031) and muscle NL (*F*_1,25_ = 23.45, *p* < 0.001). Trout from simple habitats also showed greater biosynthesis of ARA than trout from complex habitats in muscle NL (*F*_1,25_ = 6.02, *p* = 0.021) (Figure 6D).

## 4 Discussion

Using the new method of fatty acid‐specific stable hydrogen isotope analysis, a clear compensatory mechanism based on n‐3 LC‐PUFA biosynthesis and routing was demonstrated, potentially offering protection against neural impairment under a diet deprived of n‐3 LC‐PUFA (Lund et al. 2012). However, our results also clearly show that an n‐3 LC‐PUFA‐deprived diet is suboptimal for brain development, for which there are three main indicators: (1) n‐3 LC‐PUFA‐deprived trout needed to expend energy to biosynthesize DHA, shown by Δ*δ* ^2^H depletion of DHA and high ARA concentration in most tissue/lipid types; (2) n‐3 LC‐PUFA‐deprived trout needed to route DHA to the brain at the expense of muscles, shown by DHA content differing by dietary treatment in muscle tissue but not brain; and (3) n‐3 LC‐PUFA‐deprived trout had smaller brains. In short, even when the biochemical composition of brain PL was maintained through increased biosynthesis and allocation of DHA from muscle tissue, poor diets resulted in smaller brains, and trout with smaller brains performed less well in the behavioral trials.

Growth rates did not differ significantly between diet groups, which suggests that, as intended, there was no important difference in overall nutritional or energetic value between the two pellet formulae. However, it also shows, against our hypothesis, that groups raised on the low n‐3 LC‐PUFA diet did not sacrifice somatic growth to fuel the energetic demand of fatty acid biosynthesis (Tocher 2010; Závorka et al. 2021). Our study did not consider caloric intake, and it may be that *ad libitum* feeding (i.e., to satiation) allowed sufficient energy from the diet to make a sacrifice of somatic growth redundant.

Trout raised in complex habitats had smaller bodies than the trout raised in simple habitats, which appears to contradict previous findings of reduced competition via visual isolation associated with increased habitat complexity (*cf*. Sundbaum and Näslund 1997; Koljonen et al. 2012). However, the size discrepancy may be explained by a decrease in aggressive, dominant strategies in the complex habitats, as territory size and resource monopolization by dominants may be reduced (Höjesjö et al. 2004). The complex habitat tanks in this experiment were designed so that there were at least two hiding spots for each trout, minimizing the effectiveness of dominant strategies; and the weekly partial exchange of tank inhabitants occasioned the regular collapse and re‐establishment of dominance hierarchies. The lower density of trout in the complex habitats may also explain their smaller size: low stocking densities have been found in juvenile rainbow trout *Oncorhynchus mykiss* to induce chronic stress and lower feeding efficiency (Roy et al. 2021).

Sex also played a small role in size differentiation (i.e., somatic growth), which was surprising. Sex differences are normally unexpected until brown trout near maturity (Reyes‐Gavilán et al. 1997), but the juveniles of the present experiment were much younger than that. Perhaps the unlimited food resources promoted growth, which accentuated sex differences that ordinarily would not be apparent until later in ontogeny (*cf*. Rigaud et al. 2023). The relationship between sex and cognitive performance was also more pronounced in males than females. It has been observed that female brown trout are more likely than males to migrate away from complex stream habitats towards arguably simpler lacustrine, or even marine, habitats (García‐Vega et al. 2018; Huusko et al. 2017), where the advantage of such developed cognitive abilities is, perhaps, lessened. An assertion that habitat complexity can elicit a plastic developmental response may be strengthened by the interaction of sex with rearing environment in our experiment: it was the females raised in the simple habitat that had the least pronounced relationship between brain mass and cognitive performance, in addition to showing the poorest performance outright. However, the effect of sex upon any other aspect of the experiment proved minimal.

On cognitive performance, diet quality exerted its effect only indirectly. Its direct effects were on brain size and stimulation of n‐3 LC‐PUFA biosynthesis and routing to brain polar lipids, which themselves played important roles in influencing cognitive performance. In contrast, the habitats in which trout were reared resulted in direct significant differences in cognitive performance between treatment groups. Although trout raised in complex habitats showed significantly better cognitive performance than those raised in simple habitats, as predicted, this was not because their brains were larger; nor did they show significantly different percentages of either EPA or DHA (except in muscle NL). Habitat complexity did not appear to stimulate biosynthesis of n‐3 LC‐PUFA, or their preferential allocation to the brain. We suggest that constant exposure to habitat complexity during ontogeny may continually reinforce interactions between existing neurons without requiring n‐3 LC‐PUFA for the formation of new neurons (*vide* Dorman et al. 2018).

Although nursery habitat played no role in encephalization, counter to our prediction, the effect of diet followed the predicted pattern previously observed among wild brown trout: those trout with access to greater proportions of n‐3 LC‐PUFA in their diet had significantly larger brains than their lower dietary n‐3 LC‐PUFA counterparts (*cf*. Závorka, Lovén Wallerius, et al. 2022). However, save one exception, there was no difference in the relative size of any specific brain region, including optic tectum or telencephalon, between treatment groups, contrary to our prediction. We suspect that n‐3 LC‐PUFA routed to the brain was distributed proportionately to brain regions, but our study design, which analyzed lipids in the whole brain, was unable to determine fatty acid contents of individual regions. The exception was the olfactory bulb, which showed an interaction effect of diet and habitat. Presumably, there is an advantage in complex habitats to having heightened processing abilities of olfactory cues, although this may be less important than other brain functions and, so, may be sacrificed when trout are subjected to n‐3 LC‐PUFA scarcity. However, when raised in a simple habitat, it remains a mystery why trout fed a low n‐3 LC‐PUFA diet should have larger olfactory bulbs than those fed the n‐3 LC‐PUFA‐enriched diet.

As predicted, trout with larger brains showed significantly better cognitive performance than smaller‐brained trout in a task requiring ecologically important competences of recognition, memory, and inference to de‐escalate conflicts (Drew 1993), in line with our prediction. However, no individual brain region had an effect on cognitive performance. Brain regions in teleost fishes are each involved in a variety of specific cognitive functions from learning and engagement in complex social tasks (telencephalon) through processing primary visual input (optic tectum) to spatial orientation and proprioception (cerebellum) (Kotrschal and Kotrschal 2020). We suggest that all these functions may be needed together to confer the cognitive abilities required by the environments presented in this study. Therefore, total brain size was a better predictor of cognitive performance than any particular brain region.

This study found evidence that deprivation of dietary n‐3 LC‐PUFA stimulated trout to biosynthesize EPA and DHA from precursor fatty acids (such as ALA; *vide* Appendix A, Figure A2). Converting short‐chain to long‐chain PUFA as a likely compensatory mechanism has been previously established in experimental rats (Rapoport and Igarashi 2009; Rapoport et al. 2010) and humans (Barceló‐Coblijn and Murphy 2009; Domenichiello et al. 2015), reminiscent of patterns seen in the present study. Significant depletion of *δ* ^2^H in EPA and DHA in the trout raised on the low n‐3 LC‐PUFA diet compared to those raised on high n‐3 LC‐PUFA, without significant differences in the percentage of total brain polar lipids composed of those n‐3 LC‐PUFA, suggests compensation for deficiency in the diet.

The increased percentage of the n‐6 LC‐PUFA, ARA, across all tissue/lipid types among the trout fed low n‐3 LC‐PUFA may appear to be an overcompensation. Omega‐6 PUFA and ARA in particular are important for wound healing, inflammation, coagulation, and osmoregulation (Castro et al. 2016), although they can also have negative effects by increasing the risk of hyperinflammation (Layé 2010). The abundance of ARA in n‐3 LC‐PUFA‐deprived trout is more likely to be merely a consequence of bioconversion. Neither ALA 18:3n‐3 nor LIN 18:2n‐6 can be synthesized *de novo* by vertebrates and must be obtained from food sources (Blondeau et al. 2015; Malcicka et al. 2018). However, they compete for the same elongases and desaturases to perform endogenous conversion to respective n‐3 and n‐6 LC‐PUFA (Sprecher 2000). Although n‐3 fatty acids have been observed to be the preferred substrates for desaturase activity (Jeromson et al. 2015; Nakamura and Nara 2004), this is not absolute; the conversion of n‐6 fatty acids has been seen, at least in zebrafish ( *Danio rerio* ), to occur in a ratio to n‐3 of *ca*. 1:2.5 (Hastings et al. 2001). Therefore, the relative abundance of biosynthetic ARA found in trout raised on the LC‐PUFA‐deprived diet provides additional evidence of compensatory biosynthesis of DHA (and EPA).

**FIGURE A1:**
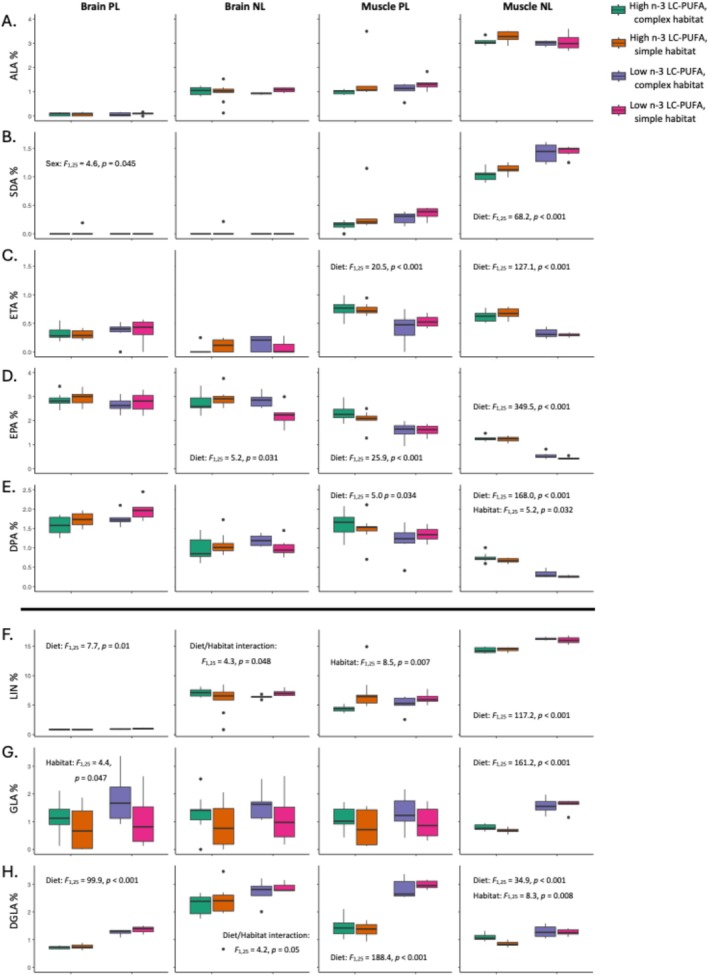
Effects of diet, habitat, and sex, for each of four tissue/lipid sample types, on the percentage of total lipids composed of (A) ALA 18:3n‐3, (B) SDA 18:4n‐3, (C) ETA 20:4n‐3, (D) EPA 20:5n‐3, (E) DPA 22:5n‐3, (F) LIN 18:2n‐6, (G) GLA 18:3n‐6, and (H) DGLA 20:3n‐6. Significant effects are noted. PL, polar lipids; NL, neutral lipids.

**FIGURE A2:**
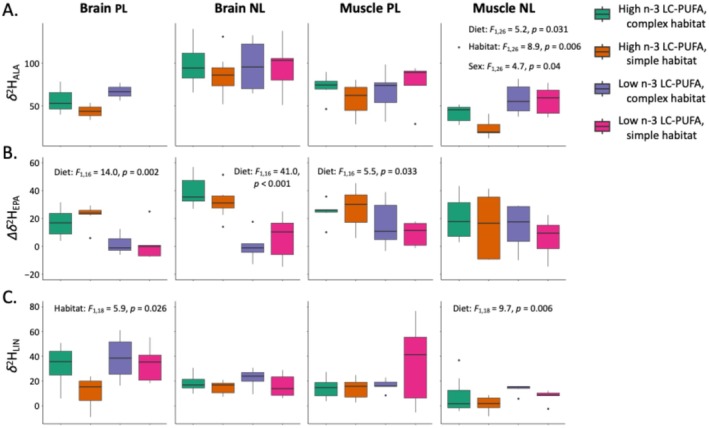
Effects of diet, habitat, and sex, for each of four tissue/lipid sample types, (A) *δ* ^2^H of ALA 18:3n‐3, (B) *Δδ* ^2^H of EPA 20:5n‐3, and (C) *δ* ^2^H of LIN 18:2n‐6. Significant effects are noted. PL, polar lipids; NL, neutral lipids.

Furthermore, the distribution of fatty acids among various tissue/lipid samples, particularly the increasing percentages of longer chain PUFA in brain PL (n‐3) or muscle PL (n‐6), suggests routing priorities (Lacombe et al. 2018; *vide* Appendix A, Figure A1). Faced with a deficiency in dietary n‐3 LC‐PUFA, trout routed available DHA (and EPA) away from muscle tissue, where it might promote hyperplasia and muscle fibre development (Wang et al. 2020), to brain polar lipids, where it might help maintain neural function, and hence cognition (Pilecky et al. 2021; Závorka et al. 2023). Trout raised on a low n‐3 LC‐PUFA diet appear to have used a combination of biosynthesis and priority routing of LC‐PUFA for active use in membranes (PL) at the expense of triacylglycerol storage (NL) to compensate for dietary lack. The compensation was, however, not complete: despite similar fatty acid composition in brain PL between the two diet treatment groups, the low n‐3 LC‐PUFA diet still resulted in smaller brains.

We are aware of the resemblance between our ‘simple habitat’ and typical fish‐farm conditions, which may point to implications for aquaculture practices. Fish fed a diet deficient in n‐3 LC‐PUFA were able to compensate in part through nutrient routing and biosynthesis, perhaps providing evidence for the practicality of non‐fish‐oil‐based diets in aquaculture, when fed to satiation. The potential benefits of habitat enrichment are more complicated, however: the improved cognitive performance of fish raised in the complex experimental habitats may be considered an advantage from the perspective of animal welfare, but this appears to come at the cost of diminished growth.

A limitation of the study was that all fish were fed *ad libitum*. With no curtailment of the amount of energy or precursor short‐chain PUFA available, the compensatory effects of LC‐PUFA biosynthesis and routing under a suboptimal LC‐PUFA deprived diet in the present study are likely to be exaggerated. In nature, where *ad libitum* feeding is not observed, we expect differences in fatty acid profiles between natural diet groups to be more pronounced with attendant ramifications for brain morphology and cognitive ability (Závorka, Lovén Wallerius, et al. 2022).

Although we raised subjects from egg, our analyses were based on data collected at a particular point in development. How well the patterns described by our findings hold throughout development, or even when in early ontogeny they first become apparent, is currently unknown and needs to be elucidated by future research. Future research should sample at multiple points throughout ontogeny. Although the age of our specimens resulted in brain masses that were too small for individual brain regions to be used for compound‐specific SIA, older specimens with larger brains could be used to shed light upon regional fatty acid distribution. Additionally, further detail of the spatial distribution of lipids within brain regions, even in fish as young as our specimens, could be advanced through the employment of new matrix‐assisted laser desorption/ionization–mass spectrometry imaging (MALDI–MSI) (Pilecky, Fink, et al. 2023). Our study was further limited by using brain mass as a proxy for neuron number. It is the number and density of neurons and their connections that determine information processing capacity (Kverková et al. 2022), and the effect of DHA on neuron proliferation and as a prophylactic against degradation has been established, at least experimentally, in model vertebrate species (e.g., Katakura et al. 2013; Lo Van et al. 2019). However, future research may combine compound‐specific SIA with a recent methodological breakthrough to quantify neuron numbers and scale with brain mass (Marhounová et al. 2019).

The alternative diets upon which trout were raised proved to be the most important differentiator of treatment groups in this study. Diet exercised clear effects on brain development, cognitive abilities, and LC‐PUFA biosynthesis and routing of brown trout, and effected a divergence in the fatty acid profiles of muscle tissue. The potential decrease in the production of n‐3 LC‐PUFA by primary producers due to climate change appears, based on the results of this study, to presage profound changes to the behavioral ecology of stream‐dwelling fishes such as brown trout. Although the extent of these changes has yet to be determined, this study makes clear that a diet bereft of adequate n‐3 LC‐PUFA is suboptimal. In wild settings, where fish do not feed *ad libitum*, the effects of n‐3 LC‐PUFA deprivation are likely to be more severe. Furthermore, the complexity of nursery habitat also plays an essential role, independently of diet quality, in the development of cognitive skills. Therefore, further studies are needed that integrate consideration of life history and diet in wild animals. Whether the divergence observed in this study is substantial enough to play a role in the development of the morphological and life‐history variants observed in wild populations of brown trout deserves study (Závorka, Lovén Wallerius, et al. 2022).

## Author Contributions

**J. Peter Koene:** conceptualization (lead), data curation (lead), formal analysis (lead), investigation (lead), methodology (equal), project administration (lead), writing – original draft (lead), writing – review and editing (lead). **Libor Závorka:** conceptualization (supporting), methodology (equal), writing – review and editing (equal). **Matthias Pilecky:** formal analysis (supporting), methodology (equal), writing – review and editing (equal). **Kathryn R. Elmer:** conceptualization (supporting), resources (supporting), writing – review and editing (equal). **Colin E. Adams:** conceptualization (supporting), resources (lead), supervision (lead), writing – review and editing (equal).

## Conflicts of Interest

The authors declare no conflicts of interest.

## Data Availability

All data and code used in the analyses are publicly accessible at https://doi.org/10.6084/m9.figshare.28282034.

## Appendix A

### Establishment of Trial Duration

To determine an appropriate duration for behavioural trials, in which dyads would consistently form stable dominant/subordinate relationships, a preliminary test, using the same protocol as the observer trials, had established 5 h to be a reasonable period in which to assess the development of dominant/subordinate relationships: of 27 preliminary size‐matched dyads, 26 formed a clear dominant/subordinate relationship after 5 h (*χ* ^2^ = 23.15, df = 1, *p* < 0.001, showing a goodness‐of‐fit departure from a 50:50 ratio of clear to unclear hierarchies), with a mean time ± SD to detection by the observing researcher of 3 h 21 min ± 54 min. The dyad was left in the test tank for a further 24 h after the end of the trial. The stability of the dyad relationship was then re‐tested by 5 min observation of behaviour followed by confirmatory bloodworm feeding. The re‐test showed that most (24 of 26) dominant/subordinate relationships remained stable (*χ* ^2^ = 18.62, df = 1, *p* < 0.001, showing a goodness‐of‐fit departure from a 50:50 ratio of hierarchies that remained the same after 24 h to those that became unclear or changed). Specimens used in the preliminary trials were not used again in the main behavioural trials.

### Routing of ARA 20:4n‐6

Distribution of ARA differed by tissue/lipid type (*F*_3,114_ = 80.1, *R* ^2^_adj_ = 0.67, *p* < 0.001), with a higher percentage used in polar lipids (*post hoc*: muscle PL vs. all other types *p* < 0.001; brain PL vs. other types *p* < 0.001, but lower than in muscle PL). Muscle NL had, comparatively, the lowest amount of ARA (*post hoc*: vs. all other tissue/lipid types *p* < 0.001) (Figure 6C).

**TABLE A1:** Ingredients of contrasting experimental diets produced by GARANT, Austria, with nutritional information and mean percentages of total lipids composed of individual n‐3 and n‐6 fatty acids as indicated by the producer.

	Feed components	High LC‐PUFA diet	Low LC‐PUFA diet
Ingredients	Fish meal, Super prime, 67% XP	20	—
	Sunflower protein concentrate, 45% XP	10	10
	Blood meal SD	7	7
	Haemoglobin powder	3	3
	Poultry meal	14.8	20
	Wheat gluten, 80% XP	2	8.5
	Soy protein concentrate	—	2.9
	Soybean meal XP	—	5
	Wheat, feed quality	13.4	10.9
	Wheat feed flower	10	10
	Fish oil	7.9	—
	Rapeseed oil	10.9	14.8
	Lin oil	—	5
	Monocalciumphosphate	—	1.1
	Lysine‐HCl	0.06	0.69
	Methionine	0.22	0.4
	Threonine	0.09	0.24
	Premix	0.6	0.6
Nutrition	Digestible energy, MJ	20.3	20.3
	Crude protein, %	42	42
	Crude fat, %	23	22
	Crude fibre, %	1.4	1.5
	P, %	1.06	1.05
	Lysine, %	2.65	2.65
	Methionine, %	1	1
	Meth + Cys, %	1.45	1.5
	Threonine, %	1.6	1.6
n‐3 fatty acids	α‐Linolenic acid (ALA) 18:3n‐3, %	4.81	5.25
	Stearidonic acid (SDA) 18:4n‐3, %	—	—
	Eicosatetraenoic acid (ETA) 20:4n‐3, %	—	—
	Eicosapentaenoic acid (EPA) 20:5n‐3, %	3.69	0.9
	Docosapentaenoic acid (DPA) 22:5n‐3, %	—	—
	Docosahexaenoic acid (DHA) 22:6n‐3, %	4.95	0.89
n‐6 fatty acids	Linoleic acid (LIN) 18:2n‐6, %	15.77	18.38
	γ‐Linolenic acid (GLA) 18:3n‐6, %	—	—
	Dihomo‐γ‐linolenic acid (DGLA) 20:3n‐6, %	0.04	0.17
	Arachidonic acid (ARA) 20:4n‐6, %	0.38	0.14

**TABLE A2:** One‐way ANOVA testing differences in means of the percentage of total lipids composed of individual n‐3 and n‐6 FAME across four tissue/lipid types; where significant, Tukey's HSD was used to test pairwise differences between tissue/lipid types. (Data shown in Figure 6 and Figure A1).

ANOVA					Tukey's HSD				
	Sum sq.	Mean sq.	*F*_3,114_	Pr(< *F*)		Difference	Lower	Upper	*p*_adj_
ALA 18:2n‐3	143.50	47.83	543.90	< 0.001	Brain PL:Brain NL	−0.924	−1.127	−0.721	< 0.001
Residuals	10.03	0.09			Muscle NL:Brain NL	2.102	1.901	2.303	< 0.001
					Muscle PL:Brain NL	0.186	−0.015	0.388	0.080
					Muscle NL:Brain PL	3.026	2.825	3.227	< 0.001
					Muscle PL:Brain PL	1.110	0.909	1.312	< 0.001
					Muscle PL:Muscle NL	−1.916	−2.115	−1.716	< 0.001
SDA 18:3n‐3	29.75	9.92	453.30	< 0.001	Brain PL:Brain NL	−0.001	−0.102	0.100	1.000
Residuals	2.49	0.02			Muscle NL:Brain NL	1.215	1.114	1.315	< 0.001
					Muscle PL:Brain NL	0.258	0.158	0.358	< 0.001
					Muscle NL:Brain PL	1.216	1.115	1.316	< 0.001
					Muscle PL:Brain PL	0.259	0.158	0.359	< 0.001
					Muscle PL:Muscle NL	−0.957	−1.056	−0.857	< 0.001
ETA 20:4n‐3	5.15	1.72	61.83	< 0.001	Brain PL:Brain NL	0.248	0.134	0.362	< 0.001
Residuals	3.16	0.03			Muscle NL:Brain NL	0.429	0.316	0.542	< 0.001
					Muscle PL:Brain NL	0.557	0.443	0.670	< 0.001
					Muscle NL:Brain PL	0.181	0.068	0.294	< 0.001
					Muscle PL:Brain PL	0.301	0.196	0.422	< 0.001
					Muscle PL:Muscle NL	0.128	0.015	0.240	0.019
EPA 20:5n‐3	67.18	22.40	132.70	< 0.001	Brain PL:Brain NL	0.094	−0.188	0.375	0.822
Residuals	19.23	0.17			Muscle NL:Brain NL	−1.784	−2.063	−1.505	< 0.001
					Muscle PL:Brain NL	−0.767	−1.046	−0.488	< 0.001
					Muscle NL:Brain PL	−1.877	−2.156	−1.598	< 0.001
					Muscle PL:Brain PL	−0.960	−1.139	−0.581	< 0.001
					Muscle PL:Muscle NL	1.017	0.740	1.293	< 0.001
DPA 22:5n‐3	30.58	10.19	29.97	< 0.001	Brain PL:Brain NL	0.881	0.482	1.281	< 0.001
Residuals	38.78	0.34			Muscle NL:Brain NL	−0.508	−0.904	−0.112	0.006
					Muscle PL:Brain NL	0.379	−0.017	0.775	0.066
					Muscle NL:Brain PL	−1.389	−1.785	−0.993	< 0.001
					Muscle PL:Brain PL	−0.502	−0.898	−0.106	0.007
					Muscle PL:Muscle NL	0.887	0.494	1.280	< 0.001
DHA 22:6n‐3	16,127	5376	239.6	< 0.001	Brain PL:Brain NL	20.354	17.111	23.597	< 0.001
Residuals	2558	22			Muscle NL:Brain NL	−9.592	−12.808	−6.376	< 0.001
					Muscle PL:Brain NL	13.778	10.562	16.995	< 0.001
					Muscle NL:Brain PL	−29.946	−33.162	−26.730	< 0.001
					Muscle PL:Brain PL	−6.575	−9.792	−3.359	< 0.001
					Muscle PL:Muscle NL	23.370	20.182	26.559	< 0.001
LIN 18:2n‐6	3099.9	1033.3	541.7	< 0.001	Brain PL:Brain NL	−5.731	−6.677	−4.785	< 0.001
Residuals	217.5	1.9			Muscle NL:Brain NL	8.471	7.533	9.408	< 0.001
					Muscle PL:Brain NL	−0.924	−1.862	0.013	0.055
					Muscle NL:Brain PL	14.201	13.264	15.139	< 0.001
					Muscle PL:Brain PL	4.806	3.869	5.744	< 0.001
					Muscle PL:Muscle NL	9.395	−10.325	−8.465	< 0.001
GLA 18:3n‐6	0.53	0.18	0.39	0.758	*Not applicable*				
Residuals	51.31	0.45							
DGLA 20:3n‐6	66.95	22.32	80.11	< 0.001	Brain PL:Brain NL	−1.864	−2.288	−1.441	< 0.001
Residuals	43.63	0.38			Muscle NL:Brain NL	−1.745	−2.165	−1.325	< 0.001
					Muscle PL:Brain NL	−0.815	−1.235	−0.395	< 0.001
					Muscle NL:Brain PL	0.119	−0.301	0.539	0.882
					Muscle PL:Brain PL	1.049	0.629	1.469	< 0.001
					Muscle PL:Muscle NL	0.930	0.514	1.347	< 0.001
ARA 20:4n‐6	114.14	38.05	80.11	< 0.001	Brain PL:Brain NL	0.216	−0.256	0.688	0.633
Residuals	54.14	0.47			Muscle NL:Brain NL	−1.009	−1.477	−0.541	< 0.001
					Muscle PL:Brain NL	1.719	1.251	2.187	< 0.001
					Muscle NL:Brain PL	−1.225	−1.693	−0.757	< 0.001
					Muscle PL:Brain PL	1.503	1.036	1.971	< 0.001
					Muscle PL:Muscle NL	2.728	2.264	3.192	< 0.001

**TABLE A3:** Results from MANOVA testing effects of diet, habitat and sex on the percentage of total lipids composed of individual n‐3 and n‐6 FAME from four tissue/lipid types. (Data shown in Figure 6 and Figure A1).

		Pillai	*F*_1,25_ ^a^	Pr(< *F*)
ALA 18:3n‐3	Diet	0.148	0.96	0.451
	Habitat	0.246	1.79	0.166
	Sex	0.088	0.53	0.716
	Diet:Habitat	0.176	1.18	0.348
SDA 18:4n‐3	Diet	0.747	16.20	< 0.001
	Habitat	0.232	1.66	0.194
	Sex	0.205	1.42	0.261
	Diet:Habitat	0.061	0.36	0.834
ETA 20:4n‐3	Diet	0.849	31.00	< 0.001
	Habitat	0.070	0.41	0.797
	Sex	0.128	0.81	0.532
	Diet:Habitat	0.233	1.67	0.193
EPA 20:5n‐3	Diet	0.942	89.78	< 0.001
	Habitat	0.145	0.93	0.465
	Sex	0.141	0.91	0.478
	Diet:Habitat	0.069	0.41	0.802
DPA 22:5n‐3	Diet	0.911	55.98	< 0.001
	Habitat	0.215	1.50	0.236
	Sex	0.020	0.11	0.978
	Diet:Habitat	0.222	1.57	0.218
DHA 22:6n‐3	Diet	0.965	153.34	< 0.001
	Habitat	0.380	3.34	0.027
	Sex	0.041	0.23	0.917
	Diet:Habitat	0.122	0.76	0.560
LIN 18:2n‐6	Diet	0.859	33.42	< 0.001
	Habitat	0.506	5.64	0.003
	Sex	0.062	0.37	0.830
	Diet:Habitat	0.219	1.54	0.226
GLA 18:3n‐6	Diet	0.950	103.53	< 0.001
	Habitat	0.207	1.43	0.256
	Sex	0.212	1.48	0.242
	Diet:Habitat	0.319	2.58	0.066
DGLA 20:3n‐6	Diet	0.909	54.89	< 0.001
	Habitat	0.339	2.83	0.049
	Sex	0.017	0.10	0.983
	Diet:Habitat	0.316	2.54	0.069
ARA 20:4n‐6	Diet	0.730	13.52	< 0.001
	Habitat	0.166	0.99	0.435
	Sex	0.335	2.52	0.073
	Diet:Habitat	0.091	0.50	0.735

^a^ Degrees of freedom for ARA 20:4n‐6 are 1 and 23 for each variable.
